# Analysis of composite endpoints in gene expression studies in oncology

**DOI:** 10.1186/1753-6561-9-S1-A17

**Published:** 2015-01-14

**Authors:** ZA Marhoon, SM Borgan, K Zakeri, LK Mell

**Affiliations:** 1 Royal College of Surgeons in Bahrain, P.O. Box 15503, Adilya, Bahrain; 2 University of California San Diego, U.S.A; 3 Department of Radiation Oncology. University of California San Diego, USA

## Background

Event-free survival (EFS) endpoints are composite endpoints frequently used in cancer gene expression studies to evaluate the effects of gene expression on patient outcomes. Event free Survival endpoints in oncology, such as Overall survival, typically combine both cancer-specific ‘death from cancer’ and non-cancer specific events ‘death from other causes’. Reporting analysis on each event comprising the composite endpoint is necessary to draw more specific inferences regarding outcomes, especially in the presence of competing risks. The extent to which cancer-specific and non-specific events are separated in contemporary gene expression studies in oncology is unknown.

## Methods

We identified 259 gene expression studies published between June 2007 and January 2012, with analysis of at least one EFS endpoint. We excluded meta-analyses (n=14), studies in recurrent/metastatic disease (n=22,) studies without EFS endpoints and studies that censored competing events (n=39), studies in foreign languages (n=4), retracted, irrelevant to research topic or unavailable online (n=22). The remaining 158 studies were independently evaluated by two reviewers according to the extent of reporting on each of the events comprising the EFS endpoint.

## Results

Sixteen studies could not be categorized because endpoints such as EFS were undefined or ambiguously defined. Of the remaining 142 studies, fifteen (10.6%; 95% confidence interval (CI), 5.4-16.2%) reported effects on both cancer and non-cancer events comprising the EFS endpoint. None of these reported any statistical analysis. Forty-Two studies (29.6%; 95% CI, 21.1-35.9%) reported only the effect on the cancer-specific component of endpoints, but not its complement, with statistical analysis provided in 18 (12.7%; 95% CI, 6.8-19.3%). In eighty-five studies (59%; 95% CI, 50.4-69.3%), no effects on cancer-specific components of the EFS endpoints were given.

## Conclusions

The majority of gene expression studies do not report cancer-specific effects comprising the Event Free Survival endpoints. Increased specificity is required in the design and reporting of cancer gene expression studies.

